# Supplementary material for: COVID-19 Vaccine Intention among Rural Residents in Mexico: Validation of a Questionnaire
Source: Vaccines (Basel). 2021 Aug 26;9(9):952. doi: 10.3390/vaccines9090952 (PMC8473309; doi:10.3390/vaccines9090952)
Supplement: Supplementary file 1 [file vaccines-09-00952-s001.zip › vaccines-1317745-supplementary/Documents_S1-S2.pdf]

S1: Cuestionario final en español (Final questionnaire in Spanish)

Sección 3: Percepción positiva de la vacunación en general

		Muy en desacuerdo	En desacuerdo	Ni de acuerdo ni en desacuerdo	De acuerdo	Muy de acuerdo
**18.**	**Las vacunas son seguras.**	1	2	3	4	5
**19.**	**Confío en el consejo del personal médico y de enfermería cuando me dicen que tengo que vacunarme.**	1	2	3	4	5
**20.***	**Las vacunas no funcionan.**	1	2	3	4	5
**21.***	**Las vacunas contienen ingredientes dañinos para la salud.**	1	2	3	4	5
**22.***	**Las vacunas causan efectos negativos a la salud a largo plazo.**	1	2	3	4	5

Sección 4: Percepción positiva de la vacuna contra COVID-19

		Muy en desacuerdo	En desacuerdo	Ni de acuerdo ni en desacuerdo	De acuerdo	Muy de acuerdo
**23.**	**Si me aplico la vacuna contra el COVID-19, el riesgo de contagiarme por COVID-19 va a bajar.**	1	2	3	4	5
**25.**	**Aplicarme la vacuna contra el COVID-19 es una forma de proteger a la gente que me rodea del COVID-19.**	1	2	3	4	5
**27.**	**Vacunarnos todos en contra del COVID-19 es necesario para terminar con la pandemia de COVID-19 en México.**	1	2	3	4	5
**28.**	**En cuanto tenga la oportunidad, me vacunaré contra el COVID-19.**	1	2	3	4	5

Sección 5: Percepción negativa de la vacuna contra COVID-19

		Muy en desacuerdo	En desacuerdo	Ni de acuerdo ni en desacuerdo	De acuerdo	Muy de acuerdo
**29.**	**No me vacunaré contra el COVID-19 porque muy probablemente tendré una reacción alérgica o efecto secundario grave.**	1	2	3	4	5
**30.**	**No me vacunaré contra el COVID-19 porque los efectos secundarios no me dejarán ir a trabajar.**	1	2	3	4	5
**32.**	**No me vacunaré contra el COVID-19 porque la vacuna no es segura.**	1	2	3	4	5
**33.**	**No me vacunaré contra el COVID-19 porque creo que la vacuna no sirve.**	1	2	3	4	5
**34.**	**No me vacunaré contra el COVID-19 porque no cuento con información suficiente respecto a la vacuna.**	1	2	3	4	5
**35.**	**Creo que no me voy a contagiar por COVID-19, por lo que no necesito aplicarme la vacuna.**	1	2	3	4	5

Sección 6: Creencias personales como barrera de salud

		Muy en desacuerdo	En desacuerdo	Ni de acuerdo ni en desacuerdo	De acuerdo	Muy de acuerdo
**37.**	**Rezar me ha funcionado para prevenir que me contagie por COVID-19.**	1	2	3	4	5
**38.**	**Si Dios quiere, me mantendré sano.**	1	2	3	4	5
**39.**	**Si me enfermo, es por la voluntad de Dios.**	1	2	3	4	5
**40.**	**De algo me tengo que morir.**	1	2	3	4	5
**41.**	**Me voy a enfermar sin importar lo que haga.**	1	2	3	4	5

* Estos ítems deben ser codificados de manera inversa en la base de datos, ya que tienen una dirección opuesta al sentido del resto de ítems de la sección.
(* These items have to be coded in the opposite direction as they are answered, so that they keep the same sense among the other of items in the section.)

S2: English translation of the final questionnaire

Section 3: Positive perception of vaccination

		Strongly disagree	Disagree	Neither agree, nor disagree	Agree	Strongly agree
**18.**	**Vaccines are safe.**	1	2	3	4	5
**19.**	**I trust in the advice of the medical and nursing staff when they tell me I need to get vaccinated.**	1	2	3	4	5
**20.***	**Vaccines do not work.**	1	2	3	4	5
**21.***	**Vaccines contain ingredients that are harmful to health.**	1	2	3	4	5
**22.***	**Vaccines cause long-term negative health effects.**	1	2	3	4	5

Section 4: Positive perception of the COVID-19 vaccine

		Strongly disagree	Disagree	Neither agree, nor disagree	Agree	Strongly agree
**23.**	**If I get vaccinated against COVID-19, my risk of getting COVID-19 will decrease.**	1	2	3	4	5
**25.**	**Getting the COVID-19 vaccine is one way to protect the people around me from COVID-19.**	1	2	3	4	5
**27.**	**Vaccinating us all against COVID-19 is necessary to end the COVID-19 pandemic in Mexico.**	1	2	3	4	5
**28.**	**As soon as I have the opportunity, I will get vaccinated against COVID-19.**	1	2	3	4	5

Section 5: Negative perception of the COVID-19 vaccine

		Strongly disagree	Disagree	Neither agree, nor disagree	Agree	Strongly agree
**29.**	**I will not get vaccinated against COVID-19 because I will most likely have an allergic reaction or serious side effect.**	1	2	3	4	5
**30.**	**I will not get vaccinated against COVID-19 because the side effects will prevent me to go to work.**	1	2	3	4	5
**32.**	**I will not get vaccinated against COVID-19 because the vaccine is not safe.**	1	2	3	4	5
**33.**	**I will not get vaccinated against COVID-19 because I think the vaccine is useless.**	1	2	3	4	5
**34.**	**I will not get vaccinated against COVID-19 because I do not have enough information regarding the vaccine.**	1	2	3	4	5
**35.**	**I do not think I am going to get COVID-19, so I do not need to get vaccinated.**	1	2	3	4	5

Sección 6: Personal beliefs as a health barrier

		Strongly disagree	Disagree	Neither agree, nor disagree	Agree	Strongly agree
**37.**	**Praying has helped me to prevent myself from getting COVID-19.**	1	2	3	4	5
**38.**	**If it is God's will, I will stay healthy.**	1	2	3	4	5
**39.**	**If I get sick, it is by the will of God.**	1	2	3	4	5
**40.**	**I will die of something.**[1]	1	2	3	4	5
**41.**	**I'm going to get sick no matter what I do.**	1	2	3	4	5

* These items have to be coded in the opposite direction as they are answered, so that they keep the same sense among the other of items in the section.)

[1] This item refers to the cultural belief in Mexico, that it is not worth to adopt healthy attitudes or preventive measures towards diseases, since we will all die eventually of a disease. This is the literal English translation from the Spanish, since there is no similar saying in the English language and is probably not applicable for English-speaking participants.
