# Supplementary material for: COVID-19 Vaccine Intention among Rural Residents in Mexico: Validation of a Questionnaire
Source: Vaccines (Basel). 2021 Aug 26;9(9):952. doi: 10.3390/vaccines9090952 (PMC8473309; doi:10.3390/vaccines9090952)
Supplement: Supplementary file 1 [file vaccines-09-00952-s001.zip › vaccines-1317745-supplementary/Document_S5.pdf]

Medicina y Asistencia Social, A.C.
Licencia Sanitaria 12046600010
Amapolas 99
Col. Aviación. C.P. 41304
Tlapa de Comonfort, Gro.

CARTA DE CONSENTIMIENTO INFORMADO

Tlapa de Comonfort, Gro., a______de________________de 20_____

Nombre completo del paciente

____

Usted es candidato(a) a participar en el protocolo de investigación "Prevalencia de aceptación de la comunidad rural hacia la vacuna contra COVID-19 en la región de la Montaña del Estado de Guerrero". El estudio se llevará a cabo en las instalaciones de Medicina y Asistencia Social, A.C, con el respaldo del Dr. Héctor Salvador Álvarez Manzo (C.P. 12098830). El protocolo de investigación fue aprobado previamente por el Comité de Investigación y el Comité de Ética en Investigación de Médica Sur. Si usted acepta participar en el estudio, es importante que considere la siguiente información. Siéntase libre de preguntar cualquier asunto que no le quede suficientemente claro.

El objetivo del estudio es determinar la prevalencia de aceptación y rechazo de la población adulta hacia la vacuna contra COVID-19, así como la percepción del riesgo de esta enfermedad, la percepción de la vacunación en general y contra COVID-19, además de la relación de las creencias personales que puedan influenciar en la toma de decisión respecto a la aplicación de la vacuna contra COVID-19 en las personas que habitan en la región de la Montaña de Guerrero y que son atendidos en la Clínica +MAS. Para esto, es necesario haber leído y firmado el presente consentimiento informado, y someterse a un cuestionario.

PARTICIPACIÓN VOLUNTARIA Y RETIRO: Su participación es voluntaria y gratuita. Lea cuidadosamente este documento antes de decidir participar en el estudio y pregunte cualquier duda a quien le está invitando a este protocolo de investigación. Puede negarse o retirar su consentimiento en cualquier momento sin que esto afecte su acceso a los servicios disponibles en la Clínica +MAS.

RESPONSABILIDADES.
La responsabilidad de los participantes incluye, pero no se limita a la siguiente: proporcionar información completa y veraz.

PROCEDIMIENTOS DEL ESTUDIO
En la primera visita, se invita al paciente candidato a participar en el protocolo, se explican el objetivo del estudio y los requisitos de participación, se recaba por escrito el consentimiento informado, se entrega una copia del consentimiento informado y se realiza un cuestionario de datos sociodemográficos y para la evaluación de la percepción de la población rural mexicana respecto a la vacuna contra COVID-19. En caso de ser necesario, se le invitará en una segunda ocasión de 1 a 2 semanas después con el objetivo de aplicar nuevamente el cuestionario.

En la misma visita se realiza un cuestionario sobre la percepción de riesgo hacia la enfermedad por COVID-19, fuentes de información de COVID-19, percepción de la vacunación en general, percepción de la vacuna contra COVID-19, y la percepción de las creencias personales y como se relacionan estás con la aplicación de la vacuna contra COVID-19.

BENEFICIOS POTENCIALES: Este estudio no está diseñado para beneficiarle directamente, ni recibirá remuneración o beneficio monetario por su participación en el estudio. Sin embargo, si acepta participar estará colaborando en la investigación. Además, su participación beneficiará indirectamente a otras personas al permitir evaluar la percepción general de la población ante la vacunación contra COVID-19 y un eventual impacto en la estrategia de vacunación.

RIESGOS: Su participación en el estudio no implica ningún riesgo.

CONFIDENCIALIDAD Y PROTECCIÓN DE DATOS: Toda la información que proporcione para el estudio será considerada estrictamente confidencial y será accesible únicamente para el personal capacitado involucrado directamente con este estudio. Quedará identificado(a) con un número y no con su nombre o cualquier otro dato que pueda identificarle. Los resultados del estudio serán publicados con fines científicos o técnicos, pero la información se presentará de tal manera que usted no podrá ser identificado(a).

AVISO DE PRIVACIDAD Simplificado: El investigador principal de este estudio, es responsable del tratamiento y resguardo de los datos personales que nos proporcione, los cuales serán protegidos conforme a lo dispuesto por la Ley General de Protección de Datos Personales en Posesión de Sujetos Obligados. Los datos personales que le solicitaremos serán utilizados exclusivamente para las finalidades expuestas en este documento. Usted puede solicitar la corrección de sus datos o que sus datos se eliminen de nuestras bases o retirar su consentimiento para su uso. En cualquiera de estos casos le pedimos dirigirse al investigador responsable.

Medicina y Asistencia Social, A.C.
Licencia Sanitaria 12046600010
Amapolas 99
Col. Aviación. C.P. 41304
Tlapa de Comonfort, Gro.

Si usted tiene alguna pregunta, comentario o preocupación con respecto a la campaña, por favor comuníquese con el Dr. Rafael Cardoso Arias, Director Médico de Medicina y Asistencia Social, A.C. al teléfono 5624499336 en un horario de 09:00 a 16:00 horas de lunes a viernes, o envíe un correo electrónico al correo rcardoso@mas.org.mx

Declaración de la persona que da el consentimiento

- Se me ha dado a leer esta carta de consentimiento informado.
- Me han explicado el protocolo incluyendo el objetivo, los posibles riesgos y beneficios, y otros aspectos sobre mi participación.
- Se me ha comunicado el derecho a cambiar mi decisión en cualquier momento antes de cualquier intervención involucrada en este protocolo.
- Me comprometo a proporcionar información completa y veraz, así como seguir las indicaciones médicas con el propósito de que mi atención sea adecuada.
- He podido hacer preguntas relacionadas a mi participación en el protocolo, y me han respondido satisfactoriamente mis dudas.

Si usted entiende la información que le hemos dado en este formato, está de acuerdo en participar en este protocolo de manera total y también está de acuerdo en permitir que su información de salud sea usada como se describió antes, por lo que le pedimos que indique su consentimiento para participar en este protocolo. Ante dos testigos y el médico que otorga información y recaba el consentimiento, firmo o imprimo mi huella dactilar como constancia de mi aceptación voluntaria en protocolo de investigación "Prevalencia de aceptación de la comunidad rural hacia la vacuna contra COVID-19 en la región de la Montaña en el Estado de Guerrero", el día____del mes de ________ del 20____en Tlapa de Comonfort, Gro.

Investigador principal: Dr. Héctor Salvador Álvarez Manzo (C.P. 12098830)
Dirección del investigador: Department of Molecular Microbiology and Immunology, Bloomberg School of Public Health, Johns Hopkins University, 615 N Wolfe St, Baltimore, MD 21205, USA
Contacto del responsable de la clínica: Dr. Rafael Cardoso Arias, tel. celular 5624499336. Correo electrónico: rcardoso@mas.org.mx
Investigadores participantes: Dr. Rafael Badillo Dávila
Nombre del patrocinador del estudio: No contará con patrocinio.
Versión del consentimiento informado y fecha de su preparación: Versión 2, 19 de febrero de 2021.

¿Lee y entiende bien el español (castellano)?	SI	NO	¿Cuál es su lengua materna o nativa?
¿Necesita de un traductor?	SI	NO	—

______________________________<br>Nombre completo y firma del paciente/familiar/tutor o representante legal	______________________________<br>Nombre completo y firma del delegado que otorga información y recaba consentimiento
______________________________<br>Nombre completo y firma de testigo<br>Relación o parentezco con paciente:______________	______________________________<br>Nombre completo y firma de testigo<br>Relación o parentesco con paciente:______________
